# Prospects for finding the mechanisms of sex differences in addiction with human and model organism genetic analysis

**DOI:** 10.1111/gbb.12645

**Published:** 2020-02-11

**Authors:** Udita Datta, Sarah E. Schoenrock, Jason A. Bubier, Molly A. Bogue, James D. Jentsch, Ryan W. Logan, Lisa M. Tarantino, Elissa J. Chesler

**Affiliations:** ^1^ Center for Systems Neurogenetics of Addiction, The Jackson Laboratory Bar Harbor Maine; ^2^ Center for Systems Neurogenetics of Addiction, Department of Genetics University of North Carolina at Chapel Hill Chapel Hill North Carolina; ^3^ Center for Systems Neurogenetics of Addiction, Psychology State University of New York at Binghamton Binghamton New York; ^4^ Center for Systems Neurogenetics of Addiction, Psychiatry University of Pittsburgh School of Medicine Pittsburgh Pennsylvania

**Keywords:** addiction, addiction predictive, animal models, behavior, genetics, genomics, heritability, neurobiological, sex differences, substance use disorder

## Abstract

Despite substantial evidence for sex differences in addiction epidemiology, addiction‐relevant behaviors and associated neurobiological phenomena, the mechanisms and implications of these differences remain unknown. Genetic analysis in model organism is a potentially powerful and effective means of discovering the mechanisms that underlie sex differences in addiction. Human genetic studies are beginning to show precise risk variants that influence the mechanisms of addiction but typically lack sufficient power or neurobiological mechanistic access, particularly for the discovery of the mechanisms that underlie sex differences. Our thesis in this review is that genetic variation in model organisms are a promising approach that can complement these investigations to show the biological mechanisms that underlie sex differences in addiction.

## Graphical abstract

Sex differences in addiction are well documented in prevalence, trajectory and treatment outcomes. Human genetic studies require tremendous sample sizes to identify the biological basis of these effects. Control over environmental influences and drug exposure combined with the ease of neuro chemical and neuro anatomical access make model organisms especially suited to reveal the mechanisms underlying sex differences in addiction.

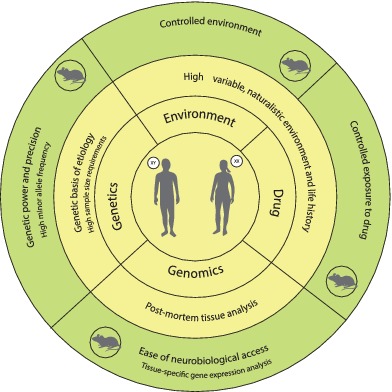

## 1 INTRODUCTION

Sex and gender differences in many aspects of drug use, drug effect and substance use disorders (SUDs) are well‐documented, but the biological mechanisms underlying these differences and their implications for risk, prevention and treatment are poorly understood. Although human genetic studies remain the standard for establishing genetic etiology for a complex neuropsychiatric condition like addiction, studies attempting to investigate sex‐specific differences in processes of addiction largely lack the phenotypic breadth, power and neural tissue access needed to discover the underlying molecular mechanisms. In contrast, genetic studies in model organisms benefit from lower sample size requirements, and a wealth of in vivo technologies for research into mechanisms. Model organism studies have showed or corroborated observations of sex and gender differences in addiction. Therefore, there is potential for genetic mapping of these phenomena to identify mechanisms of sex differences in addiction.

In this review, we highlight the epidemiological evidence for sex differences in SUDs, the influence of the environment on such sex differences and the promise and challenge of genetic analysis for discovering the biological mechanisms that underlie sex differences in addiction through the use of human and model organism discovery genetics. We highlight the major gender differences in phenomena related to SUDs, and the progress made toward studying these phenomena in human genetic and conventional model organisms studies. The literature on these phenomena is quite uneven with respect to the classes of drugs investigated across species and genetic backgrounds but show some general phenomena across classes of drugs. In other cases, the magnitude and direction of effects vary across strains and species. We have chosen examples from the literature that reflect this diversity.

Overall, few human genetic studies have succeeded in identifying sex‐specific loci for addiction because of inadequate statistical power. Although human genetic studies have been attempted for each major class of drugs, these studies are few in number and sex differences have been studied in even fewer. We have summarized these studies to call attention to this dearth of results. We argue that model organism studies show many sex differences in addiction‐related phenomena, and that with recent improvements to genetic analysis methods in model organisms, these are now amenable to genetic investigation. Model organisms have benefits of (a) increased minor allele frequency, (b) controlled genetic background variation, (c) controlled environments, (d) fully ascertained drug exposure histories and (e) access to neurobiological intermediate phenotypes that serve to increase the observed effect size of genetic loci, revealing previously undiscovered causal mechanisms underlying sex differences that can be further interrogated.

## 2 SEX AND GENDER DIFFERENCES IN ADDICTION EPIDEMIOLOGY

There are many documented differences in drug use and addiction between men and women. The Treatment Episode Data Set provided by the Substance Abuse and Mental Health Services Administration indicates sex differences in prevalence of substance use, the particular substances involved, the age at which drug use is initiated and drug use patterns.^1^ These differences are brought about by an interplay of biological traits, and social and environmental influences. Socioeconomic status, life experiences and cultural factors including societal norms and gender expectations shape drinking and drug use.^2^ According to the 2017 National Survey on Drug Use and Health, 53.6% of males and 45.7% of females 12 years or older use an illicit drug in their lifetime.^3^ Among US adults 18 years of age and older, 71.8% of males and 59.6% of females consumed alcohol within a year.^4^ Although men still consume more alcohol and illicit drugs than women, in the prevalence of substance use has become more similar across men and women over time. This has been attributed to societal factors including economic development and modernization, alcohol culture and gender equality, such that countries with greater equality show a narrowing of the gender gap in prevalence of use^5^, ^6^ and substance problem severity.^7^

## 3 GENDER DIFFERENCES IN ADDICTION TRAJECTORY

Males and females exhibit differences in the substances they use and in patterns of drug use. For example, men are more likely to smoke marijuana, while women are more likely to use alcohol and prescription drugs, including benzodiazepines and sedatives.^8^, ^9^, ^10^ Women initiate drug use at lower doses than men,^11^, ^12^ but their drug use escalates more rapidly to addiction. Women report using substances to relieve stress or negative emotions,^13^ and these negative emotions may be attributed to increased rates of sexual abuse, trauma and other stressors that are related to the cultural status of women.^14^ By contrast, men more frequently report peer influence and reinforcing properties as reasons for drug use.^15^, ^16^ Environmental risk factors contribute to addiction vulnerability differently in each sex. For instance, women who experience spousal abuse often report being coerced to use drugs/alcohol^17^ and suffer from increased risk of substance use problems.^18^, ^19^ Maternal and neonatal exposure to drugs, raise additional concerns for women and their exposed offspring.^20^, ^21^ Sexual minorities comprising the LGBTQ individuals experience higher rates of SUDs.^22^, ^23^ This is also attributed to societal environmental issues, history of traumatic life experience and other factors.^24^, ^25^, ^26^ The extent to which biological factors play an intrinsic role in substance use in these populations beyond the effects of chromosomal sex on environmental influences is unknown.

Subjective effects of drugs in women are affected by the stage of the menstrual cycle.^27^ Women have greater subjective responses to cocaine in the follicular phase of the menstrual cycle^28^, ^29^ when levels of estrogen are rising and progesterone levels are low. During the luteal phase, when progesterone levels are highest (estrogen levels are also elevated at this time), women report a reduction in the positive subjective effects of cocaine.^28^, ^29^ The physiological and positive subjective effects of cocaine are attenuated by exogenous progesterone in women, but not men.^30^ Functional magnetic resonance imaging studies indicate changes in the reward‐related neural system across the menstrual cycle and heightened reward responsivity shortly before ovulation.^31^, ^32^ Such changes in the functioning of reward‐related circuits may underlie the premenstrual increases in drug cravings.^32^ Taken together, these data highlight the importance of sex hormones in the modulation of drug effects in women.^33^

Treatment outcomes also differ between the genders. Men and women face unique challenges in cessation of drug use. Women tend to enter treatment sooner after becoming substance dependent^34^, ^35^ and present with more co‐occurring mood and anxiety disorders that complicate treatment.^36^ Women are more susceptible to craving and face a greater risk of relapse following abstinence.^37^, ^38^, ^39^

In summary, gender differences in human addiction are likely the result of sex‐specific biological mechanisms that interact with sociocultural influences and life stressors that affect individuals of different genders differently. The complex biopsychosocial interactions underlying addiction make discovery of the biological basis of sex differences in humans challenging, yet identifying these biological mechanisms is critical for the development of more precisely tailored preventative and therapeutic interventions.^40^, ^41^

## 4 HUMAN GENETIC STUDIES OF SEX DIFFERENCES IN ADDICTION

### 4.1 Twin and adoption studies

It has long been appreciated that a family history of SUD is one of the strongest risk factors for the development of drug addiction.^42^ Heritability estimates of SUDs obtained through family, adoption and twin studies^43^, ^44^, ^45^ indicate a strong contribution of genetic factors in addiction. In surveys of adult twins, heritability of addictive disorders has been estimated in a range of 0.39 for hallucinogens to 0.72 for cocaine.^46^, ^47^, ^48^

A small number of these studies have assessed the heritability of substance use by gender of the subjects^49^, ^50^, ^51^, ^52^ and interactions of gender and genetic factors were ignored because researchers typically collapsed data across drug class and gender because of small sample sizes. Heritability estimates for cocaine use disorder (CUD) have been reported to be lower in women compared with men.^52^ However, no mechanism has emerged to explain the heritability difference in CUD for males and females.^53^ Recent meta analytic studies of alcohol use disorder (AUD) indicate that gender differences are more likely attributable to social influences on alcohol exposure and use than sex‐specific genetic influences.^54^, ^55^

Genome wide linkage studies, genome‐wide association studies (GWAS), whole genome sequencing and exome sequencing have begun to identify specific genes and genetic variants that explain the heritability of SUDs, although few such studies have reported gender specific genetic effects (Table 1).

**Table 1:** Number of genome‐wide significant (*P* ≤ 5 × 10‐8) associations for various substance dependence categories including alcohol, nicotine, opioids, cocaine, methamphetamine and cannabinoids, documented on GWAS catalog (https://www.ebi.ac.uk/gwas/) as of May 12, 2019

Substance dependence	No. of significant associations	Range of effect (OR: min‐max)	Number of studies	Studies with sex‐specific analyses	Studies that reported sex‐specific associations
Alcohol	46	0.104‐19.54	15	3	2
Nicotine	6	0.032‐0.1	5	1	0
Opioids	8	0.06‐1.56	5	0	NA
Cocaine	1	Not reported	1	0	NA
Methamphetamine	3	0.104‐0.348	1	0	NA
Cannabinoids	1	1.25	1	0	NA

### 4.2 Linkage studies

Early genome‐wide linkage studies^56^, ^57^ have reported chromosomal locations harboring risk loci for cocaine,^58^ opioid,^59^, ^60^ nicotine^61^, ^62^ and alcohol dependence.^63^ As noted above, few of these historical studies have analyzed the effect of sex on genetic linkage to substance dependence. One example is a study investigating the differential risk of opioid dependence in males and females,^64^ which identified a significant sex‐specific locus associated with opioid dependence and several other suggestive loci. The positional information showed by these legacy studies can now be combined with convergent data from more recent genomic strategies to implicate genes that may play a role in sex differences in substance dependence.

### 4.3 Genome‐wide association studies

For highly complex diseases like addiction that are influenced by vast numbers of genetic variants, population‐based genome‐wide association approaches are better suited to identify risk loci with relatively small effects compared with family‐based genetic studies,^65^, ^66^ but the sample size requirements are substantial. Early studies suffered from low genetic marker density,^67^ but increased power and precision in subsequent studies has allowed identification of some causal genes.^68^, ^69^ For example, the nuclear transcription factor PKNOX2 has been identified as a sex‐specific candidate gene for composite substance dependence in women of European origin.^70^ In the absence of heroic sample sizes, the power of GWAS studies is typically insufficient to detect statistically significant associations^53^ especially in the context of sex differences. The limitations of small sample sizes have been overcome by combining GWAS data from multiple studies to allow statistical meta‐analyses.^71^, ^72^ Significant genome‐wide findings have now emerged for alcohol for example,^73^ opioids for example,^74^ cannabis for example^75^ and nicotine dependence for example^76^ (Table 1).

A recent GWAS^77^ of alcohol consumption level and AUD using the large multiancestry sample (N = 274 424) from the Million Veteran Program^78^ reported 18 genome‐wide significant loci. The sex‐stratified sampling methodology allowed detection of female‐specific signals despite the predominantly male sample, but power requirements precluded comparisons of Polygenic Risk Score by sex. In another recent GWAS analysis, a sex‐specific variant at ADGRV1 was also identified with effects on opioid dependence risk in African American males.^79^

## 5 SEX DIFFERENCES AND GENETIC MECHANISMS OF RISK FACTORS IN ADDICTION AND RELAPSE

Some GWAS studies have incorporated the stress‐related risk factors that contribute to the development of drug addiction and in addiction relapse susceptibility in GWAS. As noted above, the psychosocial and cultural factors that influence addiction epidemiology are largely attributable to stress. Stress increases vulnerability to drug addiction,^80^, ^81^ and this phenomenon differs between males and females.^82^ Females, irrespective of their drug dependency status, report significantly higher anxiety, stress and negative mood during distress^37^, ^83^ and a greater increase in drug craving as a function of stress^37^, ^84^ relative to males. Association of variants in several stress‐related genes with heroin and/or cocaine addiction have been identified in an African American sample^85^ including variants in the FKBP5 gene which contribute to the development of opiate addiction by modulating the stress response through altered glucocorticoid sensitivity.^86^ Given the importance of stress in the development and maintenance of addiction and the substantial evidence for sex differences in stress response, a thorough understanding of the sex‐specific genetic and molecular mechanisms of stress responses is essential.

## 6 SEX DIFFERENCES IN HUMAN GENOMIC STUDIES OF ADDICTION

Genomic studies examine the abundance of all expressed genes under various conditions in the postmortem human brain. The transition from recreational substance use to addiction is accompanied by drug‐induced neuroadaptations brought about by long lasting expression changes in neural genes.^87^ Gene expression profiles in individuals with a history of chronic cocaine use compared with drug naive controls have revealed a small number of genes with robust differential expression.^88^ Among the differentially expressed genes are those involved in the regulation of transcription and chromatin structure in midbrain dopaminergic neurons, highlighting the important role of epigenetic factors in drug‐induced changes in neurobiological mechanisms of addiction. Similar genome‐wide changes in midbrain gene expression have also been observed in the postmortem brains of opioid users.^88^ Given that sex differences in gene expression and splicing patterns are widespread in the adult human brain,^89^ studies of sex differences in gene expression in drug‐exposed brains could provide insight into the mechanisms of addiction, but in humans, variability of drug exposure history and the challenges inherent in postmortem brain limit the utility of this approach.

Integrating genome‐wide genetic findings with tissue‐specific gene expression genetics could reveal additional biological mechanisms underlying substance dependence. Using this approach, Huggett and Stallings have identified a SNP associated with cocaine dependence and detected three genes (two loci) underlying this predisposition that displayed robust enrichment in numerous brain regions, including the hippocampus.^90^ Gene expression genetic analyses in the GTEx project show sex differences in genetic regulation of gene expression in both reproductive and nonreproductive tissues,^91^ providing an important means of evaluating the mechanisms through which sex‐specific genetic loci might act. This approach was recently used to interpret the male‐specific effects of an opioid dependence‐associated variant.^79^

## 7 THE PROSPECTS FOR HUMAN GWAS IN THE STUDY OF SEX DIFFERENCES IN ADDICTION

In summary, human GWAS have been making inroads into the discovery of genetic influences on addiction. However, identifying the genetic components that influence sex‐specific vulnerability to addiction using GWAS has been particularly challenging because of the high heterogeneity of the population in terms of the environmental factors that influence addiction, from drug exposure and use trajectories to the involvement of interacting histories of stressful life events and other confounding influences of social, economic and cultural factors. As a result of this heterogeneity, the common variants identified for SUD in humans by GWAS have modest effect sizes. The sample size requirements necessary to achieve adequate statistical power to detect the numerous polygenic and small effect sizes associated with complex and highly heterogeneous phenotypes^92^ presents prohibitive costs and subject recruitment challenges. The sample size problem is compounded in the genome‐wide search for genetic variants associated with interacting effects of gender on addiction‐related traits.^93^ Such studies require further increases in ascertained sample sizes that can support the detection of the numerous small effects typical for complex phenotypes and their interaction with biological sex, life stress and drug exposure histories. Sample size alone cannot entirely overcome this issue, in part because the strategies used to combine studies to obtain high sample sizes often require simplification of the phenotypic data used. For most subjects, thorough histories are unavailable.

## 8 MODELING ADDICTION‐RELATED BEHAVIOR USING LABORATORY ANIMALS

Despite the inherent challenges of modeling psychiatric disorders like addiction in animals,^94^ research in model organisms present distinct advantages. Model organisms can play a vital role in identifying sex‐specific mechanisms of addiction vulnerability, trajectory and recovery through the efficient identification of pathological mechanisms, therapeutic target identification and drug development.^95^ A model is a simplification of a complex system, that is, intentionally more amenable to characterization and perturbation. Access to neurobiological intermediate mechanisms and endophenotypes, control of drug exposure history and manipulation of stress exposure provide a means of reducing noise and assessing the causal influence of environmental factors. It is, of course, essential to understand how one's model departs from the complex system that one intends to represent, and what steps one must take to transfer information from a model to the complex system. However, the benefits of efficiency, sample size, neurobiological accessibility, control over drug exposure, life‐time stress history and other details of the experimental paradigm coupled to the extensive biological, molecular and behavioral assays available in model organisms render them an important tool in the search for biological mechanisms of highly complex phenomena of addiction.

Model organisms, particularly rodents, have many conserved neurobiological and physiological features of humans and display specific facets of addiction‐related behavior and neurobiological endophenotypes. As such, they can be used to identify causal mechanisms in brain‐behavior relationships, including neurobiological and behavioral consequences of chronic substance exposure.^96^ Animal research can show specific neurobiological mechanisms (eg, molecular, cellular or pharmacological) that mediate specific aspects of addiction. A distinct advantage of animal models is that the effects of an identified mechanism can be directly tested through specific neural manipulations on processes that mediate addictive behaviors.^97^ For investigations examining the role of environmental factors on addiction, animal models offer the advantage of well‐controlled, within‐subject, longitudinal studies with significantly reduced environmental variability. In contrast, drug use in humans generally involves multiple drugs concurrently, which makes identifying the effects/interactions of specific drugs difficult.

Animal studies allow greater choice over the population's extent of genetic heterogeneity and each population has specific advantages in noise reduction for detection of small effect alleles or a broad survey of very high genetic heterogeneity with variation in nearly every gene in the genome. Minor allele frequencies in even the most complex mouse populations such as the Diversity Outbred (DO) are theoretically 12.5%, providing greater power for detection of variants that influence complex traits in substantially smaller sample sizes than required in human populations.^98^, ^99^ With the exception of large‐scale prospective studies including the Adolescent Brain Cognitive Development and All‐of‐US, studies in humans are generally limited to subjects who are already addicted, restricting the ability to separate premorbid, drug‐naïve traits from the effects of ongoing drug use. Studies in genetic reference populations of model organisms allow the assessment of multiple phases of the addiction process in genetically identical individuals, including drug‐naïve traits, initiation, maintenance, drug withdrawal and relapse. Furthermore, it is possible to execute such studies in model organisms of both sexes with identical genomes (eg, standard and recombinant inbred populations), providing ample power to detect sex‐specific and sex by genotype interactions in mechanisms and characteristics of addiction‐related traits.

## 9 INCLUSION OF BOTH SEXES IN RODENT GENETIC STUDIES OF ADDICTION

Females have been systematically understudied in neuroscience and biomedical research.^82^, ^100^, ^101^ Studying populations that include both males and females ensures that the results may generalize to other similarly diverse populations^102^ Although there is ample evidence of sex differences in drug seeking and taking, the genetic mechanisms that drive these differences remain understudied. In the Mouse Phenome Database (MPD; https://phenome.jax.org) in 2017, 47.5% of all behavioral measures are on males only, 5% on females only and 47.5% on both sexes. Strain by sex differences were observed in 42% of behavioral measures where males and females were included. Of these behavioral measures annotated to the Vertebrate Trait term “response to addictive substance trait,” 39% exhibited strain by sex differences. As this number grows, and as genetic mapping studies in these populations are performed, discovery of mechanisms of addiction‐related sex differences will be increasingly possible.

Reservations about including females have been based on the assumption that female hormonal cycles introduce substantial “noise” and complicate experimental studies relative to male‐only studies,^103^ but increased variability in females is not consistently observed.^102^ In a genetic analysis of hundreds of measures from widely used behavioral assays in BXD recombinant inbred mice, within strain variability is similar for males and females for most assays.^104^

In recent years, the number of studies that have included female subjects has increased. However, studies that have explicitly investigated sex differences^82^, ^105^, ^106^ remain limited in number and among them, only a few have conducted a rigorous test of sex differences by looking for significant interaction effects. Studying sex‐specific effects often requires large samples because interaction effects are often small. Nonetheless, a “50/50” approach^105^, ^107^, ^108^ in which males and females equally comprise each experimental group remains a prudent decision. Although such designs may not support the detection of small effect sizes, larger effects will be identified and the option of pursuing sex differences in extensions of these designs remains practical.

## 10 SEX AS A MODULATOR OF GENETIC EFFECTS UNDERLYING RODENT ADDICTION‐RELATED BEHAVIORS

There are several reviews addressing sex differences in SUD and addiction.^109^, ^110^, ^111^, ^112^, ^113^ A multitude of rodent studies of addiction‐related phenomena have evaluated drug effects across different drug classes including alcohol, opioids, psychostimulants like cocaine and amphetamines, nicotine and cannabinoids. Some aspects of sex differences are likely to be shared across all drugs, whereas others will be specific to particular substances. Addictive drugs comprise a chemically heterogeneous group with distinct molecular targets, and yet share certain characteristics. A key feature of all addictive drugs is their capacity to increase mesocorticolimbic dopamine, an action believed to be crucial for the emergence of compulsive addictive behavior^114^ albeit by different mechanisms. For example, cocaine is known to increase the extracellular levels of dopamine by inhibiting the neuronal reuptake process, whereas opioids exert their effects through activation of μ opioid receptor altering γ‐Aminobutyric acid transmission disinhibiting dopamine (DA) neurons in the ventral tegmental area which increases DA release.^115^ Sex differences have been demonstrated in drug responses across the various classes of drugs. The nature of emerging sex differences are likely to be affected by multiple factors including, the drug class, dose, developmental stage of the animal, stages of the oestrous cycle, to name a few.

Sex differences in behavioral effects of addictive drugs are widely observed, but the extent and magnitude of these differences vary across species, strains and even vendors (Table 2). For example, female rodents exhibit heightened sensitivity to psychomotor stimulant^116^, ^117^, ^118^ and reinforcing properties of cocaine.^119^, ^120^, ^121^, ^122^, ^123^, ^124^, ^125^ Various sex differences are also observed for the other drug classes under multiple behavioral paradigms.^126^, ^127^, ^128^, ^129^, ^130^

**Table 2:** Sex differences in rodent studies of addiction‐related phenomena

Assay	Author	Species	Strain/vendor	Sex difference
Sensitivity to drug effects				
Sensitivity to psychomotor stimulant effects of cocaine	Kuhn et al^116^	Rattus	Specific strain unknown, Vendor: Charles River Laboratories (Raleigh, North Carolina)	Females had enhanced locomotor response to cocaine Male and female locomotor response to cocaine was attenuated after ovariectomy/castration, but sex difference remained
	van Haaren and Meyer^117^	Rat	Male and female Wistar rats Intact and Gonadectomized Vendor: Charles River (Wilmington, Delaware)	Acute cocaine locomotor activity of intact female rats was higher than intact males and gonadectomized males and females. Only intact females displayed behavioral sensitization to cocaine after repeated drug expsoures.
	Van Swearingen et al^118^	Mus	Female C57BL/6 mice Vendor: Charles River, (Raleigh, North Carolina)	Estradiol enhanced cocaine‐induced behavior
	Zakharova et al^125^	Rat	Adolescent female and male Sprague‐Dawley rats; Socially isolated or group‐housed without or without presence or enrichment Vendor: Charles River, (Wilmington, Massachusetts)	Overall, females had increased locomotor response to cocaine. In males, cocaine response depended on social and environmental enrichment. For females, only social conditions altered cocaine‐stimulated behavior, with activity increasing with the number of rats in the cage, regardless of environmental enrichment
Reinforcing and rewarding properties of the cocaine	Hilderbrand and Lasek^119^ CPP	Mice	Male and Female C57BL/6J Vendor: Jackson Laboratory, Bar Harbor, Maine	Acquisition of cocaine CPP did not differ between male and female mice. Extinction of cocaine CPP was delayed in male mice compared with females at the lowest dose of cocaine.
	Russo et al^124^ CPP	Rat	Male and female Fischer rats; Intact and Gonadectomized Vendor: Charles River, (Kingstone, New York)	Intact females showed greater entrances (but not time spent) to cocaine‐paired side and overall locomotion. Hormones (E or E+P) can mediate reward behaviors in OVX females.
	Hu et al^120^ IVSA	Rat	Male and female adult Sprague‐Dawley rats Vendor: Harlan Sprague‐Dawley, Indianapolis, Indiana	Female rats acquired cocaine self‐administration behavior more rapidly, and they self‐administered more cocaine at a faster rate than male rats
	Jackson et al^121^ IVSA	Rat	Adult Sprague‐Dawley rats; Intact (males only) and Gonadectomized (female and male) Vendor: Harlan Inc. (Indianapolis, Indiana)	OVX+E females had increased # infusions/session, cocaine intake and acquisition (percent met criteria) at lower doses of cocaine compared with Sham and Castrated males (effects of E in females were attenuated by E+P treatment and treatment of males with E had no effect)
	Lynch^122^ IVSA	Rat	Adolescent female and male Sprague‐Dawley rats Vendor: not specified	Females had increased acquisition (percent met criteria and rate) and final ratio for cocaine infusions under progressive ratio schedule (final ratios in females also related to hormonal status)
	Lynch and Carroll^123^ IVSA	Rat	Adult male and female Wistar rats Vendor: Harlan, (Madison, Wisconsin)	Females had increased acquisition (percent met criteria and rate) and cocaine intake
	Bobzean et al^138^ CPP	Rat	Male and female Long‐Evans rats Vendor: Harlan, (Houston, Texas)	No sex differences in acquisition or extinction of CPP at multiple doses of cocaine. Females showed greater magnitude of reinstatement of cocaine CPP that is dose‐specific.
	Caine et al^139^ IVSA	Rat	Adult female and male Sprague‐Dawley rats; Intact and Gonadectomized Vendor: Charles River Laboratories (Wilmington, Massachusetts)	Intact males reached acquisition criteria (FR5 schedule) faster than females, no sex differences in dose response. No effect of gonadectomy or hormone replacement in either sex.
	Perry et al^140^ IVSA	Rat	Adult female and male Sprague‐Dawley rats Vendor: Charles Rivers (Portage, Michigan)	Most pronounced sex difference: twice as many females developed cocaine preferences (compared with natural food reward, addictive phenotype); sex differences in various types of reinstatement tests.
Genetic background x sex effects				
Sensitivity to psychomotor stimulant effects of cocaine	Zombeck et al^141^	Mice	Adolescent and adult males and females from four inbred strains: BALB/cByJ, C57BL/6J, DBA/2J, FVB/NJ Vendor: Jackson Laboratory (Bar Harbor, Maine)	Adolescents displayed reduced cocaine locomotor response as compared with adults for C57BL/6J and BALB/cByJ in both sexes while this was only observed in female FVB/NJ mice. No sex or age differences were observed for DBA/2J.
Predictors of addiction vulnerability				
Novelty‐seeking	Davis et al^162^	Rat	Sprague‐Dawley male and female rats in‐house breeding colony Bred HR and LR lines; Breeding strategy^181^	HR‐LR phenotypes predict rapidity of acquiring cocaine self‐administration HR females self‐administer more cocaine than HR males and both LR groups.
Preference for and reaction to novelty	Dickson et al^143^	Mice	Male and female DO mice (J:DO, JAX stock number 009376) from G10 to G12 from Jackson Laboratory (Bar Harbor, Maine)	Novelty‐related traits were predictive of cocaine IVSA. No main effects of sex on addiction‐related measures, effects on novelty‐related measures were observed.
Sign tracking	Dickson et al^143^	Mice	Male and female from four common inbred strains [C57BL/6J, 129S1/SvImJ, A/J, NOD/ShiLtJ] and one wild‐derived inbred strain [CAST/EiJ]	Sign‐tracking in males was genetically correlated with exploration of a novel environment, and heritability of sign‐tracking and goal‐tracking ranged from 0.32 to 0.41.
	Lovic et al^144^	Rat	Male Sprague‐Dawley Vendor: Harlan Laboratories, Indianapolis, Indiana)	STs were more impulsive on tests of impulsive action
Stress response				
Social defeat stress	Holly et al^168^	Rat	Male and female Long‐Evans Vendor: Charles River, (Wilmington, Massachusetts)	Social defeat stress resulted in behavioral and dopaminergic cross‐sensitization in both sexes, but the effect was larger and longer lasting in stressed females. Females also displayed more dysregulated cocaine taking than males, in response to stress.
Prenatal stress (PNS)	Thomas and Becker^172^	Rat	Male and female Sprague‐Dawley rats Vendor: Harlan Laboratories (Indianapolis, Indiana)	Prenatally stressed rats of both sexes exhibited more addiction‐like characteristics than nonstressed rats. Stress affects are greater in females.
	Thomas et al^173^	Rat	Male and female Sprague‐Dawley rats Vendor: Harlan Laboratories (Indianapolis, Indiana)	Exposure to PNS selectively facilitated the rate of acquisition and overall drug intake of males on an escalating‐doses regimen. Conversely, cocaine‐induced psychomotor sensitization was augmented by PNS in females, but not males.
	Bagley et al^174^	Mice	Male and female BXD strains Vendor: The Jackson Laboratory (Bar Harbor, Maine)	Strain, PNS and sex interact to modulate initial and sensitized cocaine‐induced locomotion, and CPP. QTL regulating response to PNS, and sex‐specific QTL for cocaine CPP identified.
Unpredictable chronic mild stress (UCMS)	Farhan et al^176^	Rat	Locally bred male Albino‐Wister Vendor: The Aga Khan University, Karachi, Pakistan	Male and female rats exposed to UCMS exhibited a significant decrease in cumulative food intake as well as in growth rate. Locomotor activity in home cage and open field was also decreased. Magnitude of effect was different between males and females.
	Pothion et al^177^	Mice	Males from CBA/H, C57BL/6J and DBA/2 strains Vendor: Centre Des Techniques Avancées (Orléans, France)	UCMS induced a significant decrease of the sucrose consumption in CBA/H and in C57BL/6 but not in DBA/2 mice. Impairment in long‐term spatial memory was also observed in CBA/H mice.
	Schoenrock et al^178^	Mice	Female from 37 inbred strains Vendor: The Jackson Laboratory (Bar Harbor, Maine)	Ovariectomy interacted with genetic background to alter anxiety‐like behavior.

Although the magnitude and direction of the differences may vary in different species and strains, this is a “feature,” not a “bug.” It is precisely this variation that we are harnessing in the use of genetic variation to discover the biological mechanisms of sex differences. The genes and variants detected are likely to interact with stress, as employed in the laboratory and as perceived by the subject. Stress effects vary in their direction with variation in the magnitude of the stressor. Therefore, stress‐related genes may have similar roles across species but the magnitude and direction of the effects may vary. Such was the case observed in studies of melanocortin 1 variation in mice and humans, originally detected in rodent genetic studies of stress‐induced analgesia.^131^

## 11 SEX DIFFERENCES IN TRANSCRIPTIONAL RESPONSE TO DRUGS

Gene expression analyses in reward‐related regions over time following drug exposure show mechanisms that underlie drug response, drug‐seeking and drug‐taking.^132^, ^133^ Differential gene expression analysis in the mouse nucleus accumbens before and after prolonged cocaine withdrawal have showed profound effects of sex and hormonal status in drug naïve states and fewer differentially expressed genes unique to each sex, post‐cocaine exposure.^134^ Sex‐specific differences in the brain tissue transcriptome in drug‐naive vs postdrug exposure states indicate genes that mediate sex differences in the initial response to cocaine administration and those involved in withdrawal. RNA expression differences have also been demonstrated in the NAc of adult male and female C57BL/6J mice following binge ethanol drinking sessions that was strongly influenced by sex.^135^, ^136^ Parental germline cannabinoid exposures caused stronger alterations in mRNA co‐expression patterns for synaptic plasticity genes in the dorsal striatum of female Long‐Evans rats.^137^ Identifying the molecular changes brought about by drug exposure can help identify the neurobiological substrates that are impacted in both sexes and define sex‐specific risk factors.

## 12 GENETIC EFFECTS CAN INFLUENCE THE DETECTION OF SEX DIFFERENCES

Despite the general consensus that females are more sensitive to many drug effects, some studies report either no difference^138^ or decreased sensitivity compared with males.^139^, ^140^ These disparate outcomes are caused by methodological variability and differences in age, species and strain of animals used (Table 2). Most investigations of sex differences in addiction‐related behaviors using rodents have relied on a single strain, with results that do not generalize across species or strains. The case in point is illustrated by a study of thermal nociception and morphine anti‐nociception that discovered sex differences in some strains but not others.^131^ Similar sex by genotype interactions are observed in locomotor stimulating effects of cocaine between male and female mice from genetically divergent strains^141^ and are likely to be important for other drug classes as well.^130^, ^142^ The influences of sex and genotype has also been used to investigate other addiction‐related traits. In a study with DO mice that harbor heterogenous genetic backgrounds, a sex‐specific correlation of exploratory traits to drug‐self administration was observed.^143^ In a study with the inbred founder population of DO mice, sign tracking, a trait characterized by the tendency to pursue cues that predict the reward,^144^, ^145^ was observed to be influenced by both sex and strain of the mice, and across strains males had a greater sign tracking range than females.^143^ Given the influence of genetic background on sex differences in drug response, it is possible to use genetic variation to identify the mechanisms of these differences.

## 13 GENETICS AND GENOMIC TOOLS CAN SHOW THE NATURE OF SEX DIFFERENCES IN ADDICTION‐RELATED BEHAVIOR

Rodent studies allow the evaluation of the nature of sex differences and to what extent they are attributable to chromosomal or hormonal influences. Sex differences are influenced by multiple separable and/or interacting sex‐biasing factors.^146^ Sex chromosome complement, and associated dosing of X and Y chromosome genes, is one such mechanism. These influences can be studied using the four‐core genotypes mouse model.^147^ In addition, gonadal phenotype and associated gonadal secretions, including sex steroids, can elicit both organizational effects (ie, slowly emerging and long‐lasting effects of hormones that are initiated by, but not actively maintained by, steroid levels) or activational effects (ie, steroid effects that are induced and maintained by current hormone levels). It is possible to tease these latter effects out experimentally using specialized paradigms and approaches including measurements of steroid levels, gonadectomy and hormone replacement strategies.^148^ Notably, all these mechanisms combine and interact with one another to sustain sex differences in phenotypes of interest, and once a sex difference is detected, additional studies using hormonal or chromosomal studies can provide further insight into how the genetic influence is modified by sex.

Quantitative trait locus (QTL) mapping is one of the primary genetic strategies used to show mechanisms underlying sex differences. Modern behavioral QTL studies of sex‐specific loci are readily performed in recombinant inbred mouse strains C57BL/6JxDBA/2J (BXD), experimental crosses of closely related strains (C57BL/6J (B6) and C57L/J (C57)),^149^ collaborative cross (CC) and DO mouse populations.^104^, ^150^, ^151^, ^152^, ^153^ Even though mice are unable to recapitulate the entire psychobiological diagnostic construct of addiction observed in humans, a set of alcohol and drug‐related phenotypes can be identified in mouse and humans to compare QTL data between the species.^154^ Syntenic mapping of these traits allows us to determine to what extent similar genes influence a range of drug‐related behaviors between the two species. Identification of sex‐specific loci is facilitated by the reduced environmental and genetic variability in rodent genetic studies. For example, syntenic sex‐specific QTL have been discovered that regulate alcohol consumption^155^, ^156^ and that mediate effects of opioids^157^ using rodent models.

Minor allele frequencies are typically higher in mouse populations than human populations, rendering possible the detection of small effect alleles. Selective breeding for behavioral phenotypes that correlate with drug‐seeking behaviors enriches and genetically fixes risk alleles.^158^, ^159^ In a study with high responder and low responder rats selectively bred based on exploratory locomotion in a novel environment, seven genome‐wide significant loci accounted for approximately one‐third of total variance and two‐thirds of genetic variance selected for this trait.^160^ Selective breeding has been applied to the investigation of several addiction‐related traits^114^, ^161^, ^162^ that exhibit sex differences, including alcohol consumption^161^ and cocaine self‐administration.^162^ Detection of small effect alleles, coupled to precisely defined and controlled phenotyping, allows the identification of previously unknown biological mechanisms of addiction.

Gene expression is also influenced by sex by genotype interactions, and expression QTL that represent genomic loci responsible for differential transcriptional regulation have been identified. Through correlational analysis, we have been able to move beyond sex‐specific QTL and identify sex‐specific gene expression networks.^163^ Such mechanisms of sex by genotype regulation of the molecular phenotypic variation have been observed in mental health issues in human^164^ and are expected to emerge in addiction relevant regions of the brain.^165^ High‐diversity mouse populations, such as the CC and DO populations (for review,^166^) with known and reproducible genetic variation provide a valuable platform for studying the mechanisms that drive sex dimorphic addiction‐related traits.

## 14 GENETIC ANALYSES OF SEX AS A MODULATOR IN GENE BY ENVIRONMENT INTERACTIONS

Model organisms allow the study of mechanisms of the interplay among, environmental and genetic interactions that contribute to sex differences in addiction vulnerabilities. Modulation of the interactions among stress‐ and drug‐related traits by sex has been investigated in rodents for alcohol use^167^ and other drugs.^168^, ^169^, ^170^, ^171^ Sex‐dependent outcomes of gestational (prenatal) stress augment the rewarding and neurochemical‐stimulating effects of the drug in rodents.^172^, ^173^ A recent study has identified sex‐specific QTL that modulate responsiveness to cocaine following prenatal stress in offspring of BXD recombinant inbred mice.^174^ Both sex^175^, ^176^ and strain^177^, ^178^ influence the response to stressors in the unpredictable chronic mild stress paradigm, and many sex differences in mouse behaviors are attributable to interactions with environmental variables.^179^, ^180^ Therefore, genetic studies can show mechanisms of sex differences in the stress response, and their role in addiction‐related behaviors.

## 15 CONCLUSIONS

In conclusion, genetic variation in humans and model organisms can be exploited in complementary ways to reveal the biological mechanisms that underlie sex differences in addiction. The genetic influence of sex differences in addiction‐related behavior can be detected but not readily identified in human genetic studies because of lack of statistical power at current sample sizes, and perhaps more importantly, the tremendous variability in drug exposure, lifetime history of stress and other environmental influences that contribute to human heterogeneity. However, there is now ample evidence for the existence of sex differences, and abundant evidence for genetic differences in stress‐related effects, known to often mediate or modulate sex differences in addiction‐related behaviors. Rodents exhibit many addiction‐related behaviors and sex and strain x sex differences are present in drug‐related phenotypes and predisposing traits such as vulnerability to stress effects on these behaviors. Sophisticated genetic mapping populations, neurobiological and molecular analysis tools are more readily deployed in rodent populations and sex by genotype analyses are more adequately powered as a result of the higher minor allele frequencies present in these populations. Therefore, the biological mechanisms of sex differences in many different processes of addiction are more readily discoverable using model organism genetics. The challenge remains in clearly establishing the meaning of the model—what human traits, including endophenotypes, are conserved? What elements of the biological mechanisms are conserved and which are not? Clearly, the precise genetic variants harbored by one human population or another are not readily found in a rodent population, but many elements of the molecular pathways are. These serve as valuable pointers to the mechanistic basis of sex differences in addiction and their implications for clinical applications in the prevention and treatment of SUDs.

## Data Availability

Data sharing is not applicable to this article as no new data were created or analyzed in this study.
